# Ion-Induced Polysaccharide Gelation: Peculiarities of Alginate Egg-Box Association with Different Divalent Cations

**DOI:** 10.3390/polym15051243

**Published:** 2023-02-28

**Authors:** Anastasiya O. Makarova, Svetlana R. Derkach, Tahar Khair, Mariia A. Kazantseva, Yuriy F. Zuev, Olga S. Zueva

**Affiliations:** 1 Kazan Institute of Biochemistry and Biophysics, FRC Kazan Scientific Center of RAS, Lobachevsky St., 2/31, 420111 Kazan, Russia; 2 Institute of Natural Science and Technology, Murmansk State Technical University, Sportivnaya Str. 13, 183010 Murmansk, Russia; 3 Institute of Electric Power Engineering and Electronics, Kazan State Power Engineering University, Krasnoselskaya St. 51, 420066 Kazan, Russia; 4 HSE Tikhonov Moscow Institute of Electronics and Mathematics, Tallinskaya St., 34, 123458 Moscow, Russia; 5 A. Butlerov Chemical Institute, Kazan Federal University, Kremlevskaya St. 18, 420008 Kazan, Russia

**Keywords:** sodium alginate, divalent metal cations, hydrogel, metal sorption, association structure

## Abstract

Structural aspects of polysaccharide hydrogels based on sodium alginate and divalent cations Ba^2+^, Ca^2+^, Sr^2+^, Cu^2+^, Zn^2+^, Ni^2+^ and Mn^2+^ was studied using data on hydrogel elemental composition and combinatorial analysis of the primary structure of alginate chains. It was shown that the elemental composition of hydrogels in the form of freezing dried microspheres gives information on the structure of junction zones in the polysaccharide hydrogel network, the degree of filling of egg-box cells by cations, the type and magnitude of the interaction of cations with alginate chains, the most preferred types of alginate egg-box cells for cation binding and the nature of alginate dimers binding in junction zones. It was ascertained that metal–alginate complexes have more complicated organization than was previously desired. It was revealed that in metal–alginate hydrogels, the number of cations of various metals per C12 block may be less than the limiting theoretical value equal to 1 for completely filled cells. In the case of alkaline earth metals and zinc, this number is equal to 0.3 for calcium, 0.6 for barium and zinc and 0.65–0.7 for strontium. We have determined that in the presence of transition metals copper, nickel and manganese, a structure similar to an egg-box is formed with completely filled cells. It was determined that in nickel–alginate and copper–alginate microspheres, the cross-linking of alginate chains and formation of ordered egg-box structures with completely filled cells are carried out by hydrated metal complexes with complicated composition. It was found that an additional characteristic of complex formation with manganese cations is the partial destruction of alginate chains. It has been established that the existence of unequal binding sites of metal ions with alginate chains can lead to the appearance of ordered secondary structures due to the physical sorption of metal ions and their compounds from the environment. It was shown that hydrogels based on calcium alginate are most promising for absorbent engineering in environmental and other modern technologies.

## 1. Introduction

Many ionic polysaccharides have a strong tendency to bind metal ions [1,2]. Despite the variety of structural models, many studies marked out the dominant role of ions in their triggering of the polysaccharide structural transition and subsequent aggregation of polymer chains [3,4]. Ion binding may be involved in polysaccharide gelation as a part of their biological functions and the base for numerous technological applications. Low-toxic, biocompatible and biodegradable polysaccharides have found wide applications in food technologies, cosmetology, pharmaceutical and biomedical industries [5,6,7,8]. In addition to these advanced applications, polysaccharides have been shown to be useful in the adsorption and binding of harmful chemicals, heavy metals, antibiotics, pesticides and other contaminants from water and wastewater [9,10,11,12,13,14].

**Figure 1:**
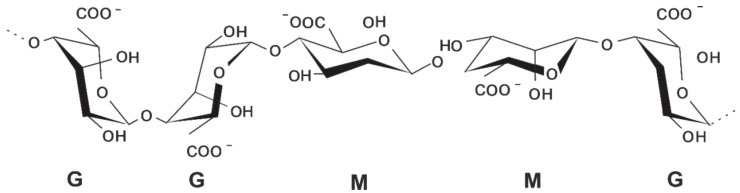
*β*–D–mannuronic (**M**) and *α–*L–guluronic (**G**) acid blocks in the alginate polymer chain.

Alginate (alginic acid) is a copolymer of *β*–D–mannuronic acid (M) and *α*–L–guluronic (G) acid (Figure 1), generally extracted from brown algae or obtained using bacterial synthesis [15,16]. In recent years, progress has been made in the large-scale bacterial synthesis of alginates with desired G and M composition and sequences [17]. Alginate belongs to a group of polymers used in food and pharmaceutical technologies as a gelling agent. It also has a strong potential in the removal of heavy metals by biosorption [18].

One of the main alginate features is its ability to undergo the sol/gel transition in the presence of bivalent cations (Mg^2+^, Ca^2+^, Sr^2+^, Ba^2+^, etc.) [19]. According to the preparation procedure, such ionotropic gels occupy an intermediate position between chemical gels with the irreversible chemical crosslinking of polymer chains by covalent bonding and reversible physical gels, where polymer crosslinking is organized by electrostatics, hydrogen bonding, chain entanglement, hydrophobic interactions and crystallization [20]. In the process of ion-induced polysaccharide crosslinking, divalent metal ions take place in the polyelectrolyte complex formation due to an electrostatic interaction between the negatively charged carboxyl groups of polysaccharide molecules and the positively charged metal cations. Such kind of interaction can lead to strong chemical bonding of cations with certain groups of biopolymers, being fundamentally different in the case of alkaline earth and transition metal cations. The properties of alkaline earth ion-induced alginate gels are close to physical gels [21,22]. On the one hand, they are thermo-irreversible, similar to covalently cross-linked gels, but on the other hand, they can be formed under mild conditions at room temperatures and physiological pH. Furthermore, slight changes in physical-chemical parameters can lead to the rapid and reversible dissolution of some ionotropic gel networks, which also makes these systems close to physical gels.

The properties of the resulting gels strongly depend not only on the polysaccharide original structure but also on the type of metal cations as crosslinking agents. The mechanism of alginate gelation, induced by divalent ions, is generally described by the egg-box model proposed 50 years ago for calcium alginate [23]. However, different types of divalent ions have their own features in the complexation with polysaccharides, giving the difference in composition and microstructure of gels [24,25,26,27,28,29,30]. For example, with the help of density functional calculations, it was shown [29] that the binding of transition metals with carboxylates and chemical interactions in cation–alginate complexes differ from those of the alkaline earth metals. These authors have shown that the complexation between alkaline earth cations and alginate units occurs only due to ionic bonds, i.e., due to electrostatic interaction. In the case of transition metal cations, the long-range electrostatic interactions compete with stronger coordination-covalent bonding of cations with alginate units [29]. Thus, two different types of interactions lead to the equal macroscopic result, which is the formation of alginate gel in the presence of metal cations due to crosslinking of polysaccharide chains.

A similar point of view was expressed in [31], where the authors considered the coordination interactions between metal ions and functional polymer groups as crosslinking junctions. Here, metal coordination employs the intermediate state between covalent and ionic bonds, being weaker than covalent bonding and stronger than ionic bonding. Thus, for different types of ions, the ion–polysaccharide system might possess properties of either physical or chemical gels, leading to the appearance of various structures with different properties.

Alginate microspheres, produced using the conventional method of ion-induced alginate gelation, in the form of a hydrogel, aerogel or xerogel, are the most popular examples of alginate usage both as an adsorbent and immobilizing carrier for enzymes [32,33], dyes [34,35] or heavy metals [36,37]. The main goal of this work is to analyze how changing the crosslinking ion affects the internal structure and sorption ability of the resulting alginate system. It has been shown that different ions lead to different types of association of alginate dimers into junction zones, which change the morphology and properties of alginate microspheres. To research the structural features of gels crosslinked with divalent metals (Ba^2+^, Sr^2+^, Ca^2+^, Zn^2+^, Cu^2+^, Ni^2+^ and Mn^2+^), we investigated the elemental composition of these gels. We have shown how information on the filling of alginate blocks by ions makes it possible to obtain information on the structure of junction zones in the hydrogel network, on the degree of filling of egg-box cells by metal cations, on the type and magnitude of the interaction between cations and alginate chains and on the most preferred types of alginate egg-box cells for cations binding and to suggest the nature of alginate dimers binding in junction zones and to analyze the presence of sorption vacancies that can be occupied by heavy metal ions. The elemental composition of metal–alginate microspheres using specific cross-linking cations has not been previously discussed in the literature.

## 2. Materials and Methods

### 2.1. Materials

Sodium alginate (A2033) from Sigma-Aldrich, USA, was used to prepare polysaccharide solutions. The chemical formula of alginic acid (C6H8O6)_n_ corresponds to both *β*–D–mannuronic (M) and *α*–L–guluronic (G) acids with M/G units ratio of 1.56 for sodium alginate [38,39].

Inorganic salts: barium chloride dihydrate (BaCl_2_·2H_2_O), strontium chloride (SrCl_2_), calcium chloride (CaCl_2_), manganese chloride tetrahydrate (MnCl_2_·4H_2_O), copper sulfate pentahydrate (CuSO_4_·5H_2_O) and zinc sulfate heptahydrate (ZnSO_4_·7H_2_O), from Tatchemproduct, Russia, and nickel sulfate hexahydrate (NiSO_4_·6H_2_O), from Sigma, were used to prepare alginate microspheres.

MQ water purified with the “Arium mini” ultrapure water system (Sartorius, Gottingen, Germany) was used to prepare all solutions.

### 2.2. Preparation of Polysaccharide Microspheres

The concentrated aqueous solution of sodium alginate (2 wt.%) was prepared according to the standard procedure [23,40,41] by dissolving polysaccharide in water, preliminary swelling at room temperature, subsequent heating to 70 °C and cooling to room temperature. Then, samples were heated and exposed to ultrasound (35 kHz, 100 W) for 60 min at a temperature of 70 °C in a water bath of Bandelin SONOREX TK52 ultrasonic disperser (Germany). To prepare microspheres of alginate hydrogel, 0.5 mL of 2 wt.% alginate solution was added dropwise under constant stirring (500 rpm) to 1.5 mL of 1M solutions of Ba, Sr, Ca, Mn, Cu, Zn and Ni salts with a medical syringe (needle diameter of 0.63 mm). When droplets of sodium alginate solution enter the salt solution, the microspheres of composite hydrogel with a diameter of about 2 mm are instantly formed, in which monovalent sodium ions are replaced by divalent metal ions. Due to ionic and donor–acceptor interactions, divalent ions bind pairwise alginate chains, leading to the formation of a three-dimensional hydrogel structure. The subsequent elemental analysis has shown that there is an almost complete replacement of sodium ions by ions of other metals. The prepared microspheres were kept in solution for 20 min, then washed twice and frozen in liquid nitrogen for freeze drying with Martin Christ equipment. The microsphere preparation procedure and washing time were the same for all samples. Images of alginate microspheres in the presence of divalent metal cations are shown in Figure S1 in the Supporting Information.

### 2.3. Scanning Electron Microscopy

Scanning electron microscopy (SEM) was performed to control the structure of the freeze-dried hydrogel microspheres. Microspheres with Ba, Ca, Cu, Zn, Ni and Mn were examined using a field emission scanning electron microscope “Merlin” (Carl Zeiss, Germany) in the Interdisciplinary Center “Analytical Microscopy” (Kazan Federal University, Kazan). Samples using Sr were studied using an instrument Auriga Crossbeam Workstation (Carl Zeiss AG, Oberkochen, Germany) in the Shared Research Center of Kazan National Research Technical University “Applied Nanotechnology” (Kazan National Research Technical University).

### 2.4. Energy Dispersive X-ray Spectroscopy

Elemental analysis was performed using energy-dispersive X-ray spectrometry (EDX) with the X-Max setup (Oxford Instruments, Abingdon, UK) combined with SEM at an accelerating voltage of 20 kV. The analytical capabilities of the Merlin field emission scanning electron microscope were extended with additional attachments for X-ray microanalysis Oxford Instruments INCAx-act with the backscattered electron diffraction (EBSD) registration system Oxford Instruments CHANNEL5. The spectrometer, thanks to the INCASynergy package, is combined with the Oxford Instruments CHANNEL5 backscattered electron diffraction (EBSD) detection and analysis system, which makes it possible to study simultaneously the distribution of elemental composition and crystalline phases in the near-surface region of the sample. Samples using Sr were studied on the Auriga Crossbeam Workstation (Carl Zeiss AG, Oberkochen, Germany), equipped with INCA X-Max silicon drift detector for energy dispersive X-ray microanalysis (Oxford Instruments, Abingdon, UK).

The energy-dispersive X-ray spectroscopy is an analytical method for elemental analysis of solid matter based on the analysis of emission energy of its X-ray spectrum. With the help of an electron beam in an electron microscope, the atoms of a studied sample are excited to emit X-ray radiation, which is characteristic of each chemical element. Investigating the energy spectrum of such radiation, one can draw conclusions about the qualitative and quantitative composition of a sample, in our case, the freeze-dried alginate microspheres. When processing the obtained results, one should take into account the existing limitations of this method associated with the absence of characteristic X-ray radiation from hydrogen and lower accuracy in determining the quantitative composition of lightweight elements, such as carbon and oxygen.

## 3. Results

### 3.1. Theoretical Background

Anionic alginate solutions can form hydrogels by using metal cations with a valence of more than one as the crosslinking agents, which can provide the coordination of polysaccharide chains via alginate carboxylate groups. The replacement of monovalent sodium by divalent metal ions Me^2+^ results in the pairwise joining of adjacent alginate chains due to the formation of metal-dependent polyelectrolyte complexes. To describe the mechanism of the alginate cross-linking by divalent Ca^2+^ ions and their further association with the formation of junction zones in the form of flat sheets, Grant et al. proposed the egg-box model [23], which was repeatedly improved [2,17,42,43,44,45,46,47]. However, with some variations, this model is still appropriate to describe the crosslinking of alginate chains with alkaline earth and transition metal ions. Subsequently, it turned out that the crosslinking of alginate chains by alkaline earth metal ions proceeds in several stages [48]. The first stage is the formation of single crosslinks between biopolymers. The diaxial bond in the homopolymeric chain of guluronates determines cavities formed by the curved fiber structure, which facilitates the metal cation accommodation inside these cavities. Since MM and MG blocks do not form such cavities, Ca^2+^ ions prefer to bind to GG blocks, although the binding to other blocks are sometimes also observed. Thus, the most optimal binding site for Ca^2+^ in the egg-box structure is the cell made up of the GG block of one chain and the GG block of an adjacent chain. At present, such an egg-box model is more often recognized as a real cell, where the ion in the cavity holds both polysaccharide chains together. Such bond formation stimulates further chain “zipping”, leading to the connection of two adjacent chains into a dimer.

**Figure 2:**
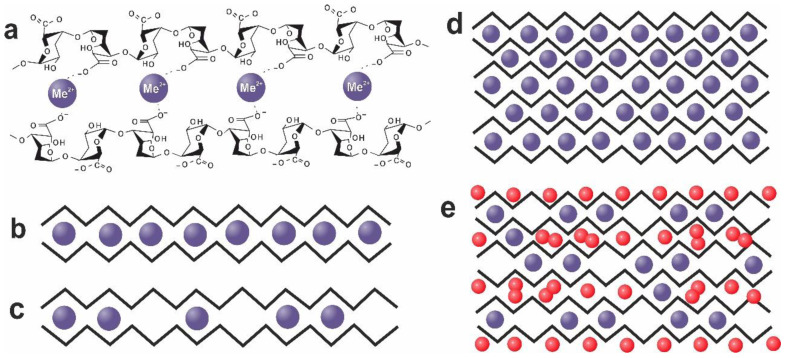
Integration of alginate chains into a dimer (**a**); schematic view of a dimer with completely (**b**) and partially (**c**) bound cells; types (**d**,**e**) of junction zones under the association of dimers. Blue balls show ions Me^2+^ and red balls correspond to sodium and hydrogen ions.

It is convenient to represent such a junction as two closely spaced alginate chains consisting of series-connected GG blocks with Me^2+^ alkaline earth metal cations included in the formed cavities, as shown in Figure 2a. The junction of all blocks leads to the formation of a completely filled dimer (Figure 2b). The presence of blocks containing M units, that are not optimal for binding leads to the formation of dimers with unbound cells (Figure 2c). Following Grant’s work [23], the alginate chains in Figure 2b,c are conventionally depicted as zigzag lines, and the absence of a bond is shown as the absence of a cation (blue ball) in the cell. Further lateral association of dimers can lead to the appearance of junction zones in the form of flat egg-box sheets. Fully cross-linked packing (Figure 2d), as will be shown later, corresponds to alginate gels induced by some transition metals, while the connected dimers bound by van der Waals interactions and hydrogen bonding are observed for calcium alginate (Figure 2e). Note that the egg-box model precisely corresponds to the scheme shown in Figure 2d. Subsequently, Sikorski [43], based on X-ray diffraction data for calcium alginate, showed that during the construction of junction zones, polymer chains are connected in the shape of already formed dimers and, therefore, half of the carboxyl groups do not participate in the bond formation. Therefore, in the case of a lateral interdimer association, the gaps between dimers contain sodium (or hydrogen) ions, which neutralize the excess charge of carboxyl groups. These ions are shown in Figure 2e as red balls. Nevertheless, the possibility of metal ions entering the zones of interdimer association exists even for calcium [28].

Since during the hydrogel formation, a divalent metal ion binds in pairs two monomer units of alginate chains, the chemical formula of sodium alginate is best considered for a block of two units as (C_12_H_14_O_12_Na_2_)_n_, or for any divalent metal Me^2+^ in the form (C_12_H_14_O_12_Me_X_)_n_, where the symbol X denotes the average number of divalent metal ions per block of two C_12_ monomeric units, i.e., the average block occupation number C_12_. The limit value X = 1 corresponds to completely filled cells of an egg-box sheet from parallel-connected alginate chains (Figure 2d). In Figure 2d, each block of two monomeric units contains a Me^2+^ ion. For dimers formed by alkaline earth metals (primarily by calcium), as shown in Figure 2e, there is one metal cation (depicted by the blue ball) per egg-box of four monomeric units. Accordingly, in the absence of metal ions in interdimer space, the average number of Me^2+^ ions per C_12_ block should not exceed 0.5. Let us recall that this case corresponds to alginate dimers bound only by van der Waals interactions and hydrogen bonding. In Figure 2e, a case corresponding approximately to X = 0.35 is shown. If metal ions also form a bond in the interdimer space, this number can increase up to X = 1. Thus, the average number of metal ions per C_12_ block obtained using elemental analysis under the use of the egg-box model provides important information about the alginate gel structure and about the type of junction zones.

In addition, it should be noted that the block occupation number should be determined by the initial composition of the studied alginate, at least by the ratio of M and G blocks in the alginate chain. The importance of determining the chain composition and sequential structure has been noted by many authors [49]. Certainly, the knowledge of alginate primary monomeric composition does not suffice to determine its sequential structure. Certain attempts to describe the probabilistic distribution of monomeric units along the polymer chain were made in [50,51,52].

To assess the possible structures of junction zones, we applied combinatorial calculus. We took into account that in the alginate sample used, the M/G ratio is 1.56 [38,39], which is close to 1.5. For simplicity, we assumed that, on average, there are three M units for every two G units, i.e., M/G = 3:2. The approximate probabilities of block formation from two monomeric units can be calculated using the variant tabulation method (see Table S1 in SI). It turned out that the probabilities of the appearance of blocks GG, GM (together with MG) and MM, respectively, are equal to 16%, 48% and 36%. Here, the GM and MG blocks were considered equivalent, although the differences between the GM–GM and GM–MG cells must be taken into account when connecting in the egg-box.

When forming the Table of variants (Table S2 in SI) for the formation of egg-box cells of various types in the space between two alginate chains, the resulting proportion GG:GM:MG:MM = 4:6:6:9 was used for subsequent comparison with the elemental experiment. The results of calculations carried out using data in Tables S1 and S2 are presented in Table 1.

In this table, cells GM–GM and MG–MG were considered equivalent to each other, but not with the cells of GM–MG (MG–GM). The remaining structures containing GM blocks or MG blocks were considered equivalent to each other. It should be noted that taking into account nonequivalent positions almost does not complicate proposed method.

### 3.2. Experimental Results

Using the field emission scanning electron microscope, the SEM images of the surface, internal sections and internal cells of the freeze-dried microcapsules, the elemental composition of the near-surface and, in some cases, internal layers of microspheres were studied and the chemical formula corresponding to this composition was determined.

Since hydrogen does not show characteristic X-ray radiation, the method of energy-dispersive X-ray spectroscopy does not allow us to determine the quantitative composition of hydrogen in the structural formula. Therefore, all further formulas are given without hydrogen, the presence of which is simply implied in the above proportions. In addition, a characteristic feature of the energy-dispersive X-ray spectroscopy method is that lightweight elements are determined with a larger error, by which we explain the systematical mismatch of oxygen atoms by almost one per one C_12_ block.

The comparative results for seven samples of metal–alginate hydrogels are given below. The sequence of samples prepared on the basis of divalent metals Ba-Sr-Ca-Zn-Cu-Ni-Mn is considered in the descending order of their ionic radii 0.135-0.113-0.099-0.074-0.073-0.070-0.067 nm [27]. Traces of aluminum present in some samples are attributed to the result of sample preparation for scanning electron microscopy.

**Figure 3:**
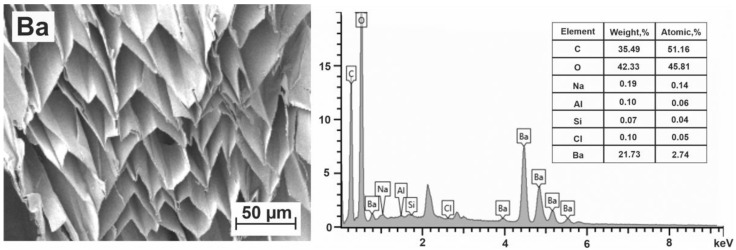
SEM image of transverse section of a Ba–alginate microsphere and its elemental content.

Barium–alginate microspheres. Cell images of the Ba–alginate microspheres and the elemental analysis data of the surface layers of freeze-dried barium–alginate microspheres are shown in Figure 3. For the above-mentioned reasons, hydrogen is absent.

The obtained elemental composition of Ba–alginate microspheres corresponds to the formula (C_12_O_11_Ba_0.6_)_n_. Here and below, the elements whose contribution is less than one tenth of an atom per cell were not included in the determined formula. For each C_12_ block composed by two monomeric units, there are on average X = 0.6 Ba atoms. This is less than the maximum possible theoretical value X = 1.0 but more than X = 0.5. There are traces of other metals (rest of Na and Al), but due to their small amount, they do not make a real contribution to a certain composition. Thus, the obtained data on the block occupation number indicate that barium cations bind alginate chains not only inside dimers but also in the interdimer space. This means that when joining in junction zones, the stage of formation of alginate dimers proceeds simultaneously with their joining into zones. Thus, in the junction zones of barium alginate, the dimers do not retain their individuality. This fact promotes the formation of strong Ba-based gels [53].

However, barium does not bind to all alginate blocks. In 40% of egg-box cells, the Ba^2+^ cross-linking cations are absent. The excess charge of the carboxyl groups remaining after sodium removal can be compensated in this case only by hydrogen. Due to the fact that, according to the available literature data, Ba interactions are more preferable with GG and MM blocks [27,28,52,53,54,55,56,57,58,59], some of the blocks, most likely MG, remain unbound by barium cations. Apparently, the shape and size of cells including MG blocks do not correspond to the barium cation. Thus, GG–GG, GG–MM, MM–MM and possibly GM–MM can be chosen as the most probable structures containing Ba^2+^. The total probability of filling the structures, equal to 385/625 ≈ 61.6%, allows us to determine the number of barium cations as X = 0.62, which is quite close to obtained value X = 0.6.

It should be also noted that after the preparation of barium alginate microspheres using barium chloride, all chloride ions remain in the washing solution after double flushing. This means that chloride ions do not interact with alginate chains. The cells of the obtained barium–alginate microspheres have a clear rhombic shape and a fairly homogeneous structure (Figure 3). The approximate cell size is 50 × 25 µm. According to [28], this gel is stable in acidic and neutral pH environments. If not for the toxicity of barium compounds, such a porous material would be ideal for drug delivery and other medical applications.

Strontium–alginate microspheres. In the table of elements, strontium occupies an intermediate position between barium and calcium. According to its properties, it is an analog of calcium. Natural strontium occurs as a mixture of four stable isotopes (mainly ^88^Sr, 82.6%). It is a constituent of microorganisms, plants, animals and, unlike barium, is a low-toxic chemical.

**Figure 4:**
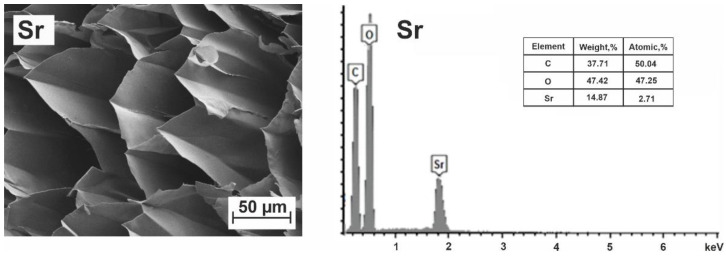
SEM image of a transverse section of a Sr–alginate microsphere and its elemental content after additional washing of the sample.

The images of cells in Sr–alginate microspheres and the data from the elemental analysis of the near-surface layers of strontium–alginate microspheres obtained by treating sodium alginate with strontium chloride are shown in Figure S1 and Figure 4. Despite the performed standard double washing, the obtained elemental composition, shown in Figure S1, corresponds to the chemical formula (C_12_O_11.6_Cl_2.3_Sr_1.8_Na_0.1_)_n_. A similar composition was obtained for the inner regions of microcapsules. A distinctive feature of the obtained formula in comparison with the theoretical composition is the presence of chlorine and a significantly larger number of strontium atoms per block, as well as the existence of sodium residues. This composition indicates the presence of structures inside the sample not inherent to strontium alginate.

The obtained formula, in which there are more strontium atoms than possible binding sites, leads to a conclusion about the existence of strontium atoms in at least two fundamentally different nonequivalent positions: (a) connecting adjacent alginate chains, i.e., acting as cross-linking agents and (b) in the composition of SrCl_2_ associates adsorbed by alginate chains. The excess amount of strontium atoms, the presence of chlorine and considerations of the electro-neutrality of the solution make one assume the formation of SrCl_2_ associates. It should be noted that SrCl_2_ structures exist precisely in the form of associates and only near alginate chains. Their possible dissociation in bulk solution would lead to the appearance of chloride ions, which are indifferent to the alginate structure and can be easily removed with washing. This is indicated by the results obtained for barium alginate gels. Such behavior of chloride ions was observed during ion-induced gelation with BaCl_2_ when the absence of chloride ions was observed after a standard double washing. Separation of contributions from nonequivalent strontium atoms shows that approximately 0.65 of crosslinking Sr^2+^ ions per each C_12_ block carry out the spatial connection of chains, leading to the formation of egg-box cells.

In addition, for each C_12_ block of strontium alginate, there are on average 1.15 associates of strontium chloride SrCl_2_. Taking into account that a distinctive feature of natural polysaccharides is the presence of sorption ability, we assumed that the binding of SrCl_2_ by hydrogel structures observed in this case (physical adsorption) is most likely due to the presence of local energetically favorable positions for these associates near the alginate chains. Despite the fact that SrCl_2_ associates do not form either covalent or ionic chemical bonds with alginate chains, they can be kept near them due to weaker (mainly van der Waals) interactions. To find the real elemental composition of strontium alginate, we subtracted 1.15 Sr atoms associated with 2.3 Cl atoms and obtained the resulting formula (C_12_O_11.6_Sr_0.65_Na_0.1_)_n_.

To test the hypothesis of the existence of various types of strontium binding with alginates, we assumed that the energy of the interaction of SrCl_2_ associated with biopolymer chains corresponds to the physical adsorption. In this case, it will be not much more than the energy of its thermal motion in water. Therefore, an increase in the washing time should lead to the removal of weakly bound SrCl_2_ associates. In the case of complexation based on ionic electrostatic interactions, the washing will not change this result.

Thus, we have conducted a study of newly prepared and thoroughly washed microcapsules of strontium alginate. The washing solution was changed five times with an interval of 2 h. The salt concentration in the wash solution was controlled using an inoLab Cond 7310 SET1 conductometer (Hungary). The washing took place at room temperature until the electrical conductivity of the washing solution reached the value of the electrical conductivity of distilled water (after 6 h of washing). The SEM images of Sr–alginate cells and elemental data for strontium–alginate microspheres after the additional washings are shown in Figure 4.

The elemental analysis carried out for two different cases of washed strontium–alginate microspheres gave new results: (a) (C_12_O_11.3_Sr_0.65_)_n_ and (b) (C_12_O_11.1_Sr_0.70_)_n_, which do not differ fundamentally from the result obtained earlier. This indicates that the used analysis of elemental composition was quite correct. In addition, this result can be regarded as an indirect confirmation of SrCl_2_ physical adsorption by the hydrogel. It should be noted that with the standard procedure of microsphere preparation, described in the Materials and Methods, the existence of associates similar to SrCl_2_ should also be expected in the case of cross-linking of alginates with other cations. It can even be assumed that the existence of such binding sites (physical adsorption) for emerging associates of metals (and other impurities) with hydrogel structure is explained by the adsorption capacity of alginate hydrogels and, therefore, can be numerically correlated with it.

According to the literature data, the structures corresponding to the egg-box model can appear as a result of the complex formation of strontium with alginate chains. Due to the known information that the interaction of strontium is more preferable with GG and MG blocks [27,28,29,58,59,60], some blocks, most likely MM, remain unbound by these cations. If we choose cells that do not contain MM blocks, namely, GG–GG, GG–GM, GM–GM and GM–MG, as the most probable structures containing Sr^2+^, we shall obtain X = 0.41. The discrepancy between this number and the experimental data means that some cells containing MM blocks also can contain Sr^2+^ cations. In particular, the occupation of structures GG–GG, GG–GM, GG–MM and GM–MM gives the total probability of filling cells equal to 400/625 = 64%. This allows us to determine the average occupation number for strontium as X = 0.64, which is close to X = 0.65 ÷ 0.7 obtained from our experiments. The experimental value X = 0.65 ÷ 0.7 cations per C_12_ block exceeds the limiting value of 0.5, corresponding to the case of the association of dimers with cations according to the type of Figure 2e. This means that, as in the case of Ba^2+^, the crosslinking occurs not only within dimers but also in the interdimer space and the dimers lose their individuality in resulting junction zones. In the case of strontium, the number of cations involved in the gel formation is still slightly larger than in the case of barium. However, a significant part of cells remains empty. Apparently, the shape and size of some cells do not correspond to the strontium cation. Thus, despite the fact that the experimental technique used does not allow us to draw conclusions about specific crosslinking sites, some considerations about the role of GG, MG and MM blocks in the binding of Me^2+^ ions by alginate chains can still be expressed.

The cells of strontium alginate microspheres obtained after intense washing turned out to be much more uniform in their composition and size (Figure 4). They are very similar in structure to the barium alginate microspheres. The Sr^2+^-based microsphere cells, which are slightly larger, also have a rhombic shape with an approximate size of about 60 × 40 µm.

Strontium alginate forms a nontoxic gel with high chemical stability and strong mechanical performance. The Sr–alginate gels show great potential as biomaterials for bone regeneration based on enhanced cell proliferation and migration. It was noted in [60] that Sr alginate is a suitable material for the immobilization of living cells in long-term perfusion studies compared with other ion-induced alginate gels. The Sr–alginate gel has higher chemical stability and effects on cells caused by Sr^2+^ are relatively mild compared to the other divalent cations.

**Figure 5:**
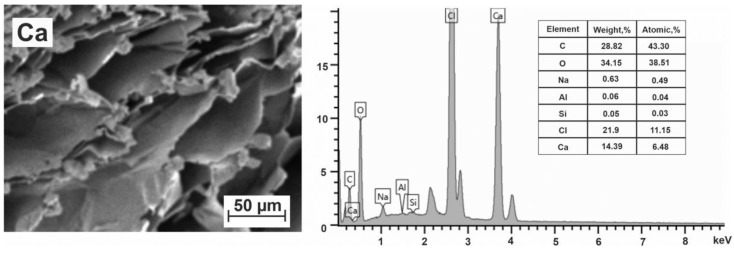
SEM image of the transverse section of a Ca–alginate microsphere and its elemental content.

Calcium–alginate microspheres. The most commonly used and studied divalent alkaline metal for the preparation of hydrogel microspheres is calcium. SEM images of the cells of the freeze-dried microspheres and data from the elemental analysis of near-surface layers of microspheres are shown in Figure 5.

The obtained elemental composition, despite the standard washing of microspheres, corresponds to the formula (C_12_O_11_Cl_3_Ca_1.8_Na_0.14_)_n_. A distinctive feature of this composition from the expected one is the presence of chlorine and a noticeably larger number of calcium atoms per block, as well as the presence of a small number of sodium atoms. Just as in the case of strontium alginate, the observed elemental composition leads to the conclusion that calcium atoms exist in two fundamentally different nonequivalent positions: (a) connecting adjacent alginate chains, i.e., acting as the cross-linking agents and (b) in the form of CaCl_2_ associates adsorbed by alginate chains. The separation of these contributions can be carried out similarly to the previous case. Assuming that the adsorption binding of CaCl_2_ associated with the hydrogel structure is due to the presence of energetically favorable local positions near the alginate chains, we subtracted 1.5 Ca atoms associated with 3 Cl atoms and obtained the following elemental composition (C_12_O_11_Ca_0.3_Na_0,14_)_n_. Thus, only 0.3 Ca^2+^ crosslinking ions in the structure of the hydrogels per C_12_ block provide spatial crosslinking of chains. This may be the reason why the strength of calcium-based gels is inferior to that of barium-based gels [53].

When carrying out theoretical calculations of the probability of the calcium cations binding in a hydrogel structure, we proceeded from the fact that bonds should occur in the cells containing GG blocks. There were no other preferences for Ca^2+^. The probability to find calcium cations in cells GG–GG, GG–GM and GG–MM is 184/625 = 29.4%, which gives X = 0.29, being close to the value of 0.3 obtained in experiment. Note that the probability of GG–GG structures all over the sample is rather small (~2.5%), but their existence triggers the zipping mechanism of chains joining into dimers. There are also not so many cells that include one GG block. Therefore, the number of crosslinking ions turned out to be very small, and the type of junction zones correspond to the lateral association of dimers due to van der Waals interactions and hydrogen bonds (Figure 2e). The probability of occurrence of calcium ions in the interdimer space is low since it is determined by the probability of contact between the GG blocks of two dimers. This probability is about 1%. The uncompensated charges of carboxyl groups are partially compensated by the presence of small amounts of Na^+^ and H^+^ ions, which are mainly concentrated in the inter-dimer space. In general, such a structural organization of calcium alginate hydrogels makes its properties closer to those of physical hydrogels. Compared with Sr^2+^, Ca^2+^ shows a smaller number of coordination sites and weaker binding with alginate molecules [29,60]. Here we are faced again with a situation where the shape and size of some cells do not correspond to the cation used. Therefore, Sr–alginate gels have a significantly higher chemical stability and stronger mechanical performance than Ca–alginate gels under the same concentration of alginate and ions. However, the total number of possible binding sites (both chemical and physical) in strontium and calcium alginates is approximately the same being about 1.8 per block of two monomeric alginate units. In comparison with strontium, the decrease in sites at which calcium ions can bind polysaccharide chains increases the number of sites suitable for physical adsorption. This fact allows us to conclude that calcium alginate can be used in the engineering of materials that are more effective in terms of sorption ability for their use in different technologies. It should also be noted that the adsorption of heavy metals by calcium alginate hydrogels leads to the strengthening of their structure [61].

The cells of calcium–alginate microspheres are heterogeneous not only in their composition but also in size (Figure 5), and their walls are rather thick compared to the walls of other metal–alginate microspheres. The approximate cell size is 70 × 30 µm. Calcium alginate gels are considered as safe and non-toxic.

Alginate systems based on transition metals Zn^2+^, Cu^2+^, Ni^2+^. The alginate microspheres with transition metals were prepared with the help of their sulfate salts obtained in contrast to the case of alkaline earth metals, for which the chlorides were used. Since the comparison of elemental composition with the participation of Me^2+^ sulfates may not always be correct due to the difference in anions, we present below only the main features that characterize these hydrogels.

Zinc, which has the largest (comparable to alkaline earth metals) ionic radius (0.074 nm) of all transition metals we studied, produces ion-induced hydrogels similar to alkaline earth metal hydrogels. It is known that the Zn–alginate gel is always loose and weak [28,29,62,63,64]. This is due to the fact that Zn^2+^ can interact with the carboxylate of G blocks in a similar way to Ca^2+^. However, the Zn^2+^-mediated cross-linking of alginate shows a unidentate binding, which involves only one carboxylate oxygen atom [28].

The obtained elemental composition of Zn–alginate microspheres made it possible to determine the average occupation number of zinc ions per C_12_ block. It turned out that, on average, X = 0.6 crosslinking Zn^2+^ ions provide a spatial connection of polysaccharide chains. This number corresponds approximately to the number of Ba^2+^ cations that create bonds in the GG and MM blocks. It is possible that zinc cations bind to the same blocks, which may explain the similar result that is obtained. The incomplete use of all possible bonds (the number of zinc ions per block is less than one) casts doubt on the fact that covalent bonds predominate over the ionic bonds [29] in zinc alginate, although it may be possible when zinc ions interact with specific blocks. The total number of possible binding sites (both chemical and physical) for this alginate hydrogel turned out to be 1.5 per block of two monomeric alginate units, which is less than the value of 1.8 obtained for the strontium and calcium systems. Perhaps this fact also determines the weak structure of zinc alginate.

The cells of zinc–alginate microspheres have a disordered structure, in which elements of tetrahedral symmetry are still visible. In general, a fairly homogeneous porous structure is observed. The approximate cell size of 30 × 30 µm is noticeably smaller than that of alkaline earth metal alginate hydrogels but still larger than that of other transition metals. In the literature, good antibacterial properties of zinc-based gels are noted, however, exceeding certain concentrations for medical purposes can lead to toxic effects. Better preservation of enzyme activity was also noted when using zinc–alginate microcapsules [62].

The mechanism of Cu^2+^- and Ni^2+^-induced gelation of alginate remarkably differs from that of the alkaline earth metals [28,29,62,65,66,67]. It was concluded that the formation of coordination-covalent bonds in alginate gels with transition metal cations apparently prevails over electrostatic interactions in polyelectrolyte solutions [29]. The ions of these transition metals bind equally well to both M and G alginate units [66], forming much more ordered structures [68,69,70]. The observed elemental composition did not give grounds for assuming that the positions of various copper ions are not equivalent. This means that all copper cations are crosslinking ones (X = 1) leading to completely ordered junction zones described by the egg-box model (Figure 2d). Despite the fact that the size of the copper cation is one and a half times smaller than the calcium one, the egg-box cell formed by alginate chains and Cu^2+^ is always longer than that formed with Ca^2+^ [28]. The increased size in the egg-box dimer formed by alginate chains and Cu^2+^ may indicate a complex association that occurs when alginate chains are bound by copper cations, including the presence of hydration water molecules in egg-box cells. The calculations of optimized structures of Cu^2+^−disaccharide complexes, which were studied with the density functional theory (DFT) [29], showed the presence of cavities that could accommodate more complicated copper complexes than single cations. Thus, it is possible that the cross-linking of alginate chains in copper–alginate microspheres and the formation of ordered egg-box structures with completely filled cells are provided by hydrated copper complexes of complicated composition.

The obtained elemental composition of Ni–alginate microspheres indicates a strong interaction of Ni^2+^ with polysaccharide chains, the average occupation number X = 1 for C_12_ blocks and the presence of hydration water which, along with the Ni^2+^ cation is responsible for the complex formation of alginate chains. In Ni–alginate microspheres, nickel ions bind equally well to all structural units of alginates, which makes it possible to speak about the formation of strong coordination-covalent bonding during complexation, leading to completely ordered junction zones in the egg-box model (Figure 2d). It should be noted that the calculations performed using the DFT method showed [29] that the presence of water molecules in the inner coordination shell of an ion provides a wide variety of stable hydrated structures. Moreover, these calculations show that the interaction energy of cations increases in hydrated complexes compared to corresponding anhydrous structures. Therefore, it is not surprising that some hydration water molecules can participate in the complex formation of alginates together with Ni^2+^.

It should be noted that many nickel compounds are toxic and carcinogenic.

**Figure 6:**
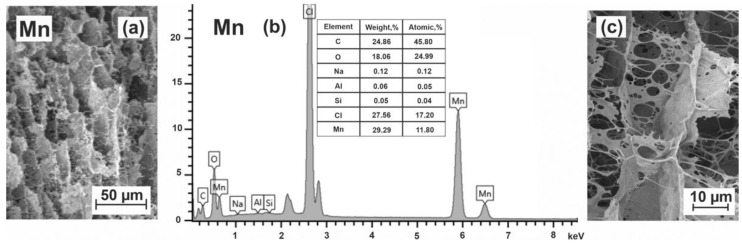
SEM image of cells in a Mn–alginate microsphere (**a**), elemental content of surface domains (**b**) and an enlarged image of cell walls of the interior (**c**).

*Manganese–alginate microspheres. SEM images of the cells and the elemental analysis data for Mn–alginate microspheres* obtained by treating alginate with manganese chloride solution are shown in Figure 6.

The resulting elemental composition of the surface layers of Mn–alginate microspheres (Figure 6b) corresponds to the formula (C_12_O_6_Cl_4_Mn_3_)_n_. A distinctive feature of this formula is an extremely small number of oxygen atoms per C_12_ block in the presence of three manganese atoms, which indicates very strong metal–alginate interactions. There was also a surprising openwork structure of the cell walls of manganese–alginate microspheres in the presence of strong Mn^2+^ interactions with all alginate blocks. Similar to calcium, we assumed that the addition of manganese salts leads not only to chain linking but also to the formation of physically adsorbed MnCl_2_ complexes. The subtraction of atoms related to MnCl_2_ associates gives the following elemental composition (C_12_O_6_Mn)_n_. The cells of each block are completely filled (X = 1) with manganese cations indicating that manganese ions form complexes equally well with all structural units of alginate chains due to the appearance of covalent-coordination bonding.

The decrease in the number of oxygen atoms requires an additional explanation. It can be assumed that the presence of manganese leads to the appearance of processes during which H_2_O and carbon dioxide CO_2_ are released. Indeed, alginic acids exhibit a number of specific chemical reactions. Such reactions include, for example, dehydrogenation, decarboxylation and degradation up to oligomers in an acidic medium [26] or on heating in the presence of some metal salts [71]. The decomposition of alginate under physiological conditions can be caused by the partial oxidation of alginate chains [72]. Apparently, the manganese compounds contribute to the occurrence of such processes, being the catalysts for many organic reactions [73]. This can be facilitated by the fact that manganese chloride MnCl_2_ is hydrated when dissolved in water, forming slightly acidic solutions with a pH of about 4. The instability of gels formed by manganese alginate has already been pointed out in [28,74].

The cells of freeze-dried Mn–alginate microspheres look very unusual. The cell partitions of the inner part of these microspheres at higher magnification are shown in Figure 6c. The openwork “petals” of cells resemble the structures left after the etching or some kind of chemical treatment, indirectly confirming by this picture the partial decomposition of alginate. According to the number of manganese atoms per block, a dense material should have been obtained, similar in properties to the nickel–alginate hydrogels. However, due to dehydration and decarboxylation, the hydrogel structure is loosened.

The approximate size of the cells can be estimated as 20 × 15 µm. The shape of the cells is closer to cubic. In our opinion, such a material could be of interest as a catalyst or catalyst carrier in biotechnological processes.

## 4. Discussion

We considered fundamental aspects of the influence of various ions on the morphology and elemental composition of alginate hydrogel microspheres obtained using various crosslinking metal ions (divalent cations Ba^2+^, Sr^2+^, Ca^2+^, Cu^2+^, Zn^2+^, Ni^2+^ and Mn^2+^). It should be noted that concentrated solutions of divalent metal salts were used to prepare hydrogel microcapsules. If the multi-staged binding of alginate chains is observed for weakly concentrated solutions [48], in highly concentrated solutions these stages occur almost simultaneously.

The result of the crosslinking of bivalent ions and polysaccharide chains is the formation of flat junction zones corresponding to egg-box structures with varying degrees of their cells being filled by divalent metal cations. The chemical formula of alginate with pairwise crosslinked chains underlying the egg-box structure, per the block of two monomers is (C_12_H_14_O_12_Me_X_)_n_. The limiting value of the average number of Me^2+^ cations per C_12_ block (occupation number) X = 1 corresponds to the case of completely filled egg-box cells. We have shown that the analysis of elemental content of the near-surface zones of metal–alginate microspheres allows us to obtain information not only on the filling degree of junction cells by metal cations but also to draw conclusions about the type and magnitude of the interaction between cations and alginate chains, to clarify information about the composition of the most preferred egg-box cells and to suggest the nature of binding of alginate dimers in junction zones. In addition, the existence of opportunities for the sorption of metal ions and their compounds by metal–alginate hydrogels, primarily by the calcium–alginate system, has been established.

A cross-comparison of the obtained results, together with an analysis of the literature data, allowed us to conclude that the average degree of cell filling X correlates with the strength of cation binding to the alginate chains, i.e., with the relative contribution of the stronger than ionic coordination-covalent interaction, which is typical for alginate in the presence of transition metal cations [29]. Therefore, for cations such as Cu^2+^, Ni^2+^ and Mn^2+^ where the contribution of the coordination-covalent interaction is sufficiently large, the average occupation number is X = 1 (Figure 6), and the junction zones have the form shown in Figure 2d. A slightly different situation is observed under the interaction of Zn^2+^, which, although a transition metal, has a larger ionic radius comparable to that of alkaline earth metals. In the case of the zinc–alginate system, the experimentally observed average occupation number turned out to be less than 1 (X = 0.6), which indicates that the bonding of zinc with alginate is rather weak and cannot be of a coordination-covalent nature. The interaction of this cation with alginate, as well as the interaction with alkaline earth metal cations, is carried out using ionic bonds. Its additional features (unidentate binding [28]) make the Zn^2+^-based hydrogel weak [62,63,64].

**Figure 7:**
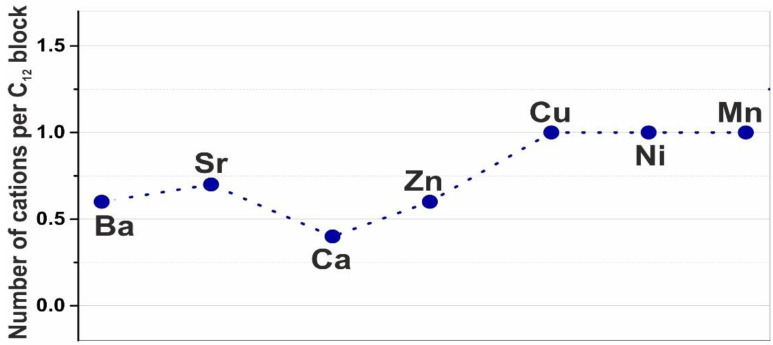
Average number of various divalent metals cations per block C_12_. Sequence of chemical elements Ba-Sr-Ca-Zn-Cu-Ni-Mn is taken in descending order of their ionic radii 0.135-0.113-0.099-0.074-0.073-0.070-0.067 nm.

The result of crosslinking with alkaline earth cations, primarily with Ca^2+^, is the pairwise association of alginate chains (chain-to-chain association) and the subsequent lateral association of cross-linked dimers, leading to the formation of flat junction zones, as shown in Figure 2e. In this case, cations fill the cells formed when alginate chains join into dimers. Due to van der Waals forces and hydrogen bonds, the dimers associate in the junction zone but there are no cations in the interdimer space. With the complete filling of all cells of each dimer with cations and their absence in the interdimer space, X = 0.5. A similar situation, but with partially unfilled cells of alginate dimers, corresponds to calcium alginate hydrogels, where the average number of crosslinking Me^2+^ ions per C_12_ block is even smaller and is approximately equal to X = 0.3. As we have shown, calcium cations can also enter the inter-dimer space, but the probability of such a process is low (about 1%). For other alkaline earth metals, the X number corresponds to 0.6 for barium and 0.65–0.7 for strontium (Figure 7). On the one hand, this fact is explained by the electrostatic binding of alkaline earth cations with alginate chains, which results in a different degree of complex formation of these cations with alginate blocks of various types (GG, MM, GM). This is manifested in a different probability of filling cells and the appearance of a certain number of unoccupied sites in the egg-box structures. On the other hand, the value X > 0.5 indicates the existence of Mn^2+^ cations in the inter-dimer association zone. Therefore, for barium and strontium, the stage of association into dimers occurs probably simultaneously with the stage of inter-dimer association, just as in the case of transition metals. The dependence, shown in Figure 7, reflects the average number of cations of various divalent metals per C_12_ block, which is equal to average occupation number in the cells of junction zones.

Molecular dynamics methods have established the existence of many optimal but still unequal binding sites for metal ions with alginate chains [17,29,75], some of which remain unoccupied. The presence of such places can lead to their occupation by various molecules which are bound by alginates as a result of physical sorption, the binding energy of which is less than the energy of the ionic bond of cations with alginate units and is comparable to the energy of thermal motion of molecules.

The interesting features of hydrogels can be revealed by studying the quantitative composition of the excess salts used to prepare microspheres and that remain in their structure after 20 min double washing. In principle, the components of the salt solution that have entered the sodium alginate solution are not equivalent. Some of the components can be incorporated into the structure of the hydrogel (Me^2+^ cations), the other part can be indifferent to gel structure and some parts can be in the zones of local energy minima, i.e., weakly interact with alginate structure. For example, when barium chloride is added, a certain amount of barium cations participates in crosslinking and all excess barium salt and chlorine anions are easily removed from the water in which hydrogel beads are located for 20 min during double washing. They are indifferent to the structure of barium alginate. On the contrary, when strontium chloride is added, almost the same amount of strontium cations is involved in crosslinking, however, not all Me^2+^Cl_2_^–^ associates were eliminated by double washing. In particular, a 6h washing was required to remove excess salts (1.15 SrCl_2_ per C_12_ block). It should be noted that washing leads to the removal of “excess” ions and associates, but not places for their possible binding, which determine the sorption capacity of alginates.

In the course of ion-induced gelation in the presence of transition metal cations, the egg-box structures of junction zones, i.e., the sheets of connected alginate chains, are formed almost instantly, so the excess salts can be located both above and below these sheets, which also form the ordered secondary structures. The elemental analysis data show that the maximum number of transition metal associates (except zinc) Me^2+^Cl_2_^–^ per C_12_ block can be 2, and their location should be determined by the properties of the metal/alginate system. Thus, the number of physically adsorbed associates in this case is even greater than in the case of alkaline earth metal alginates. It is possible that the strength of their physical binding to alginate chains will also be greater. The time of the removal of “excess” associates by washing has not yet been established.

For zinc and strontium, the number of emerging secondary associates is 0.9 and 1.15 per C_12_ block, respectively. The large size of barium ions does not allow the participation in additional interactions with alginate chains.

According to the obtained structural models, the most optimal hydrogel in terms of sorption properties is the calcium–alginate hydrogel. A small number of filled cells of the egg-box structure in calcium alginate lead to the appearance of 1.5 additional possible sites for the interaction of ions and associates with alginates per each C_12_ block. Some of these sites can be located in the inter-dimer space of junction zones, which is associated with the electronegativity of weakly filled egg-box structures. Another part may be near the alginate chains, above and below these planes. Indirectly, the appearance of secondary structures is indicated by a decrease in the level of crystallinity upon soaking a sodium alginate film in a CaCl_2_ solution, which was observed in [2].

## 5. Conclusions

In this work, we studied the elemental composition and structural features of the near-surface regions of freeze-dried microspheres obtained on the basis of the association of sodium alginate induced by divalent cations Ba^2+^, Sr^2+^, Ca^2+^, Cu^2+^, Zn^2+^, Ni^2+^ and Mn^2+^. It has been shown that in metal–alginate hydrogels, the average number of various Me^2+^ cations per C_12_ block with a limiting theoretical value equal to 1 is less than this number. In the case of alkaline earth metals and zinc, the average occupation number ranges from 0.3 to 0.35 for calcium to 0.65 to 0.7 for strontium. This fact points to the electrostatic binding of alkaline earth and zinc cations to alginate chains. A consequence of the relatively weak electrostatic interaction is a different degree in the complex formation of these cations with alginate blocks of various types (GG, MM, GM), which manifests itself in the presence of a certain number of unoccupied sites in the egg-box structure. Apparently, there is a discrepancy between the large size of the alkaline earth cation and the possibilities of its complex formation and the size and shape of certain egg-box cells. The use of combinatorial methods made it possible to calculate the probability that egg-box cells of various natures are formed during the association of alginate chains with the M/G unit ratio of 1.5.

The transition metal cations Cu^2+^, Ni^2+^ and Mn^2+^ bind alginate chains through coordination-covalent bonding, which also results in the formation of structures similar to egg-boxes but with completely filled cells, i.e., the number of cations per C_12_ block is equal to 1. However, the completely filled cells are not the only structural feature of transition metal gels. The elemental analysis shows that in the copper–alginate microspheres, the crosslinking of alginate chains and the formation of ordered egg-box structures with completely filled cells are provided by hydrated copper complexes. A similar situation is observed for the nickel–alginate hydrogels. An additional distinctive feature of manganese–alginate systems is the partial destruction of alginate chains. Thus, the metal–alginate complexes may have a more complete organization than was assumed previously.

Using the example of strontium alginates, it has been established that the existence of unequal binding sites for metal ions with alginate chains can result in the appearance of an ordered secondary structure of the hydrogel due to the physical sorption of ions and other molecules from the environment. The study of the elemental composition of excess salts remaining in the gel structure after its double washing allows us to calculate the number of sites for their possible binding, which determines the sorption capacity of alginates.

From the point of view of sorption characteristics, calcium alginate shows the most optimal properties. The reduced number of sites where calcium ions link chains increases the number of sites available for physical adsorption. In addition, during adsorption from the environment, the vacancies in interdimer space and in the egg-box structures are occupied by heavy metal cations, which, as a rule, belong to transition metals and can be intruded into cells of any configuration. Their incorporation and bonding by prepotent coordination-covalent bonding led to hydrogel structural strengthening and to the appearance of additional sites for physical sorption. Therefore, we believe that on the basis of calcium alginate, the most effective materials in terms of sorption capacity can be obtained for their use in environmental and other modern technologies. The creation of innovative nanocomposite materials with improved mechanical properties and efficient adsorption capacity [76,77] based on calcium alginate holds a great promise for various ecological purposes.

## Figures and Tables

**Table 1:** Approximate probabilities of association of M and G units into different egg-box structures for alginate with M/G~1.5.

N	Type of Structure	Probability	N	Type of Structure	Probability
1	GG–GG	16/625 = 2.56%	4	GM–GM	72/625 = 11.52%
2	GG–GM	96/625 = 15.36%	5	GM–MG	72/625 = 11.52%
			6	GM–MM	216/625 = 34.56%
3	GG–MM	72/625 = 11.52%	7	MM–MM	81/625 = 12.96%

## Data Availability

The data in this study are available on reasonable request from the corresponding author.

## Supplementary Materials

The following supporting information can be downloaded at: https://www.mdpi.com/article/10.3390/polym15051243/s1, Figure S1: Images of alginate microspheres in presence of divalent metal cations; Figure S2: SEM image of transverse section of Sr-alginate microsphere and its elemental content; Table S1: Calculation of probabilities of various blocks occurrence along alginate chain having a mannuronic acid to guluronic acid ratio M/G = 1.5; Table S2: Calculation of probabilities of various egg-box cells along alginate dimer having a mannuronic acid to guluronic acid ratio M/G = 1.5.
