# Supplementary material for: Ion-Induced Polysaccharide Gelation: Peculiarities of Alginate Egg-Box Association with Different Divalent Cations
Source: Polymers (Basel). 2023 Feb 28;15(5):1243. doi: 10.3390/polym15051243 (PMC10007407; doi:10.3390/polym15051243)
Supplement: Supplementary file 1 [file polymers-15-01243-s001.zip › polymers-2222582-supplementary.pdf]

polymers

MDPI

Supplementary Materials

Ion-induced polysaccharide gelation: Peculiarities of alginate egg-box association with different divalent cations

Anastasiya O. Makarova [1], Svetlana R. Derkach [2], Tahar Khair [3], Mariia A. Kazantseva [1,4], Yuriy F. Zuev [1,5,*] and Olga S. Zueva[3]

[1] Kazan Institute of Biochemistry and Biophysics, FRC Kazan Scientific Center of RAS, Lobachevsky St., 2/31, 420111 Kazan, Russia
[2] Murmansk State Technical University, Sportivnaya str. 13, 183010 Murmansk, Russia
[3] Kazan State Power Engineering University, Krasnoselskaya St. 51, 420066 Kazan, Russia
[4] HSE Tikhonov Moscow Institute of Electronics and Mathematics, Tallinskaya St., 34, 123458, Moscow, Russia
[5] A. Butlerov Chemical Institute, Kazan Federal University, Kremlevskaya St. 18, 420008 Kazan, Russia
* Correspondence: yufzuev@mail.ru

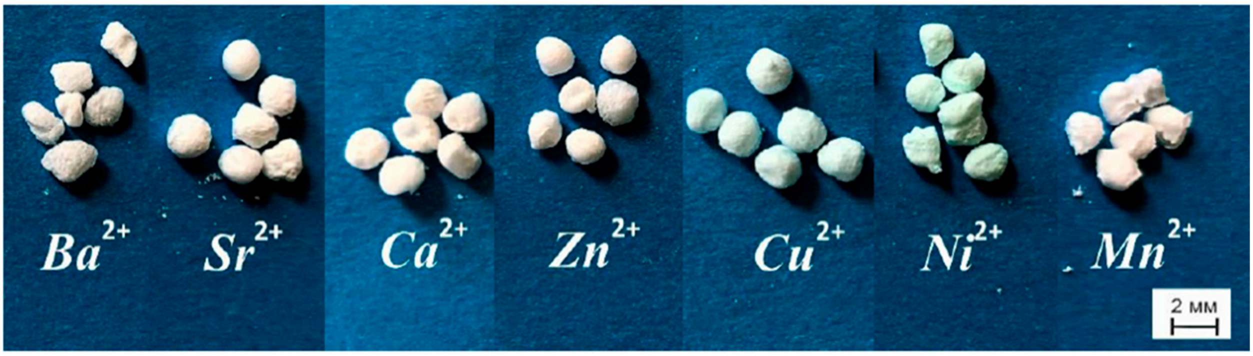

Figure S1. Images of alginate microspheres in presence of divalent metal cations.

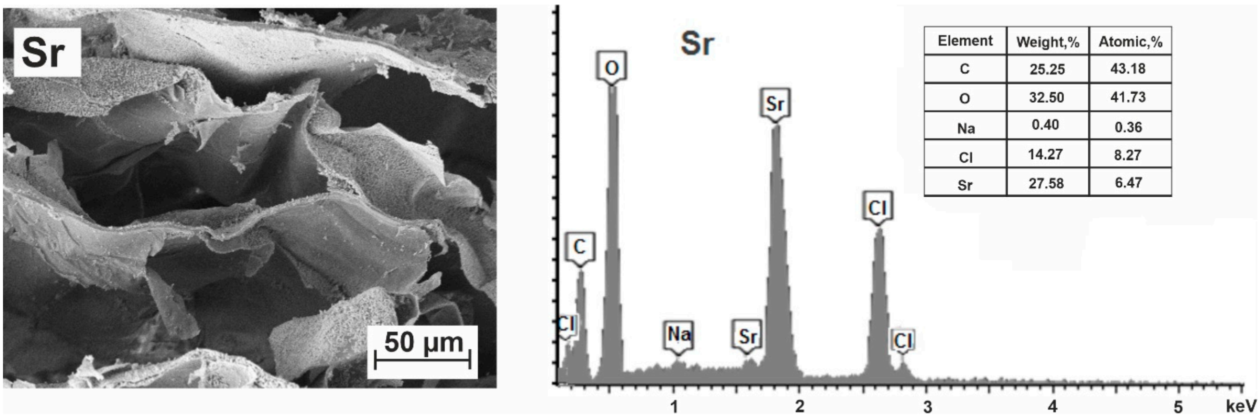

Figure S2. SEM image of transverse section of Sr-alginate microsphere and its elemental content.

Table S1. Calculation of probabilities of various blocks occurrence along alginate chain having a mannuronic acid to guluronic acid ratio M/G = 1.5.

	**G**	**G**	**M**	**M**	**M**
G	GG	GG	MG	MG	MG
G	GG	GG	MG	MG	MG
M	GM	GM	MM	MM	MM
M	GM	GM	MM	MM	MM
M	GM	GM	MM	MM	MM

Probabilities of various blocks occurrence.

GG: 4/25 = 16 %; GM: 12/25 = 48%; MM: 9/25 = 36 %.

Table S2. Calculation of probabilities of various egg-box cells along alginate dimer having a mannuronic acid to guluronic acid ratio M/G = 1.5.

	**GG**	**GG**	**GG**	**GG**	**GM**	**GM**	**GM**	**GM**	**GM**	**GM**	**MG**	**MG**	**MG**	**MG**	**MG**	**MG**	**MM**	**MM**	**MM**	**MM**	**MM**	**MM**	**MM**	**MM**	**MM**
GG																									
GG																									
GG																									
GG																									
GM																									
GM																									
GM																									
GM																									
GM																									
GM																									
MG																									
MG																									
MG																									
MG																									
MG																									
MG																									
MM																									
MM																									
MM																									
MM																									
MM																									
MM																									
MM																									
MM																									
MM																									

Probabilities of different egg-box cells.

GG-GG: 16/625=2.56 %.

GG-GM: 96/625=15.36 %.

GG-MM: 72/625=11.52 %.

GM-GM: 72/625=11.52 %.

GM-MG: 72/625=11.52 %.

GM-MM: 216/625=34.56 %.

MM-MM: 81/625=12.96 %.
